# Dutch prostate cancer patients' views about exercise and experience with exercise advice: a national survey

**DOI:** 10.1007/s11764-023-01368-3

**Published:** 2023-03-30

**Authors:** Myrthe M. Joosten, Johanna Depenbusch, Tjendo Samuel, Neil K. Aaronson, Karen Steindorf, Martijn M. Stuiver

**Affiliations:** 1 Faculty of Health, Amsterdam University of Applied Sciences, Amsterdam, The Netherlands; 2 Division of Physical Activity, Prevention and Cancer, German Cancer Research Center and National Center for Tumor Diseases Heidelberg, Heidelberg, Germany; 3 Center for Quality of Life, Netherlands Cancer Institute, Amsterdam, The Netherlands; 4 Division of Psychosocial Research and Epidemiology, Netherlands Cancer Institute, Amsterdam, The Netherlands

**Keywords:** Prostate cancer, Exercise, Patient preferences, Survey research, Barriers and facilitators

## Abstract

**Purpose:**

To support the development and implementation of exercise programming for people with prostate cancer (PC), we investigated their views on exercise.

**Methods:**

Online survey with open recruitment. We collected data on clinical and sociodemographic variables, experiences with exercise advice, outcome expectations, and preferences. We explored determinants of (1) having been counselled about exercise and (2) preferring supervised exercise.

**Results:**

The survey was completed by 171 patients (mean age = 70 years, SD = 6.5) from all PC treatment pathways. Sixty-three percent of the respondents reported never having been informed about the potential benefits of exercise. Forty-nine percent preferred exercise to be supervised. Respondents generally reported a positive attitude towards exercise. Seventy-four percent indicated barriers to exercising, including fatigue and lack of access to specific programmes. Outcome expectations were generally positive but moderately strong. Receiving hormonal therapy and younger age were significantly associated with having received exercise advice. Being insured and having higher fatigue levels contributed significantly to the preference for supervised exercise.

**Conclusion:**

Dutch people with PC report receiving insufficient effective exercise counselling. Yet, they are open to exercise and expect exercise to improve their health, although they experience various barriers that limit their ability to exercise.

**Implications for Cancer Survivors:**

The moderate outcome expectations for exercise of people with PC and their limited recall of exercise counselling highlight the need for better integration of exercise in clinical pathways. The lack of access to specific programming limits the use of evidence-based exercise programmes for people with PC.

## Introduction

Patients being treated for prostate cancer (PC), and especially those treated with androgen deprivation therapy (ADT), can experience side effects that affect their general health [1]. This includes side effects such as loss of muscle mass and strength, loss of bone mass, fatigue, reduced vitality, increased risk of falling, and changes in self-image [1–4]. Individuals with PC also often experience symptoms of anxiety and depression [5]. Consequently, many patients living with PC experience reduced quality of life [3].

There is sufficient evidence supporting the positive impact of exercise on many of these side effects and related health effects of the disease and its treatment in both the curative and palliative PC treatment settings [2, 6–10]. Exercise is planned, structured, repetitive and intentional movement intended to improve or maintain physical fitness and general health. Even though various exercise programmes have been proven to be safe and effective and are therefore recommended for men with PC [11, 12], implementation of exercise programmes in usual care for these patients is still limited [13, 14]. At the same time, the need for such programmes is growing as the incidence and survival rates of patients with PC are increasing. Even in the case of incurable disease, patients with PC can have a relatively good prognosis, with a median survival of stage IV PC (at diagnosis) of 5.5 years and a 10-year relative survival rate of 31% [15]. Higher levels of physical activity (PA; any bodily movement that requires energy expenditure) are associated with better overall and disease-specific survival [16].

Previous research has shown that a substantial proportion of men living with PC do not meet the current exercise guidelines for cancer patients, as proposed by the American College of Sports Medicine [17–19]. Also, compared to breast and colorectal cancer patients, PC patients reported the strongest decreases in PA from pre- to post-diagnosis, with significantly lower PA levels post-diagnosis (compared to pre-diagnosis) regardless of PA intensity. Likewise, the percentage of PC patients meeting PA guidelines decreased from 71 pre- to 52% post-diagnosis [20].

To improve PA behaviour and exercise uptake, awareness is key. Healthcare providers, including physicians, nurses, and nurse specialists, are adviced to talk to patients about exercising and to refer patients to available exercise programmes as part of usual cancer care [16]. Provision of exercise advice is recommended specifically for patients on ADT [21]. Patient education aimed at improving exercise behaviour should tap into patient values, expectations, and goals while also addressing strategies to overcome the most common barriers patients may face. Furthermore, to successfully implement exercise as a part of usual health care, it is important that exercise programmes themselves align with the needs of patients, their preferences, and expectations [22, 23].

Previous studies conducted in general cancer populations as well as PC populations have reported several facilitators of and barriers to taking part in exercise programmes. Important facilitators include the convenience of the programme and the belief that exercise will improve health and daily functioning, while typical barriers comprise personal factors, such as lack of motivation or perceived benefit, but also contextual factors such as costs and travel time, and structural barriers such as limited availability of specialised programmes [24–26].

These various issues are relevant to introducing broadly available exercise programming for patients with PC in the Netherlands. In the current survey, we addressed these issues as the first step in a health innovation program. The main objectives of this study were to map PC patients’ and survivors’ experiences with exercise advice and referral in the context of health care and to explore their preferences for and outcome expectations of an exercise programme after a PC diagnosis. Secondary objectives were to explore the general attitudes and perceived barriers to the exercise of Dutch men living with and beyond PC and to explore how clinical and personal characteristics (i.e., disease stage or age) might be related to receiving exercise advice and to personal preferences regarding supervised vs self-directed exercise.

## Method

### Study design

We conducted an observational, quantitative, cross-sectional study in the form of an anonymous survey with open recruitment. Data were collected between June 23rd and August 14th, 2020, in the Netherlands. The Ethics Review Committee of the Amsterdam University of Applied Sciences approved the study and affirmed that the Medical Research Involving Human Subject Act does not apply to this study. Although the survey was anonymous, respondents explicitly gave informed consent to the first question of the survey.

### Participants and recruitment

Participants were eligible for the study if they had been diagnosed with PC, regardless of treatment pathway (ongoing or completed with the intent to cure, on active treatment as part of palliative care, or on active surveillance). Eligibility was confirmed through a single question at the start of the questionnaire. Patients who were not proficient in Dutch were excluded via self-selection, as the survey was produced and available in Dutch only. Patients who did not give permission to store and use their responses for the purpose of this study were also excluded.

Recruitment took place in collaboration with the Dutch PC patient foundation (Prostaatkankerstichting, PKS). The survey was announced through their newsletter, website, and social media. The study was briefly introduced, and an open link was provided for participants to take part in the online survey.

### Survey

As per the patient foundation's advice, the survey consisted of two parts, each with a limited time burden to maximize response rates. Part one of the survey was obligatory for all participants and contained questions related to the respondent’s characteristics and key areas considered pertinent to the successful implementation of exercise programming. Sociodemographic and clinical variables included respondents’ age, geographical location, educational level, whether their insurance covered exercise programmes, year of diagnosis, and treatment type. After consulting with patient foundation representatives, disease stage was not queried directly in the survey because this was deemed unreliable and possibly emotionally confronting for the respondents. Instead, treatment intent (curative, palliative, or “watchful waiting”) was deduced by two researchers independently (MJ and MS) based on treatments received and independently validated by a nurse specialist at the Netherlands Cancer Institute. Disagreements were resolved by consensus. Experience with exercise advice and referral as part of health care were explored using several questions. Patients were asked whether they had been informed about possibilities and benefits of exercise and, if so, how (conversation, providing a booklet, internet reference, or other). A second question assessed whether patients had ever been asked about their current level of PA and by whom. Additionally, we asked whether they had been adviced to do more exercise and/or had been referred to an exercise programme at any point during or after treatment, and by whom.

Study-specific questions, developed by exercise oncology experts and epidemiologists from the Netherlands Cancer Institute and the National Center for Tumor Diseases Heidelberg, were used to assess exercise-related outcome expectations (benefits as well as risks) and corresponding values. These questions concerned muscle strength, bone strength, pain, fatigue, mood, sleep, and self-esteem. Items were measured on a 0–10 numeric rating scale. For outcome expectations, this was linearly converted to a range of − 5 to 5, with negative scores indicating negative outcome expectations and positive scores indicating positive expectations. For the perceived value of these outcome expectations, 0 represented “totally unimportant” and 10 represented “very important”. For each outcome, the expectation score was multiplied by the corresponding value score. These multiplied scores were then summed, resulting in an overall value*expectation score with a possible range from − 50 to + 50.

To indicate preferences for exercise delivery, companions with whom to exercise, supervision, location, frequency of exercise, time willing to travel, and willingness to pay, the most preferred option could be selected from a predefined list. For preferences regarding the type of exercise and physical activities, respondents could choose three activities that appealed to them the most. For all preference questions, respondents could also choose “no preference” or “I don’t know”. Respondents were asked to indicate how long (per session) they thought they would be able to exercise and their preferred intensity (low, moderate, or high intensity, each of which was defined; e.g. moderate intensity was explained as “noticeably increased heart rate and breathing frequency, but still able to talk”). Since data was collected during the COVID-19 pandemic, we explicitly asked the respondents to “answer the questions based on your situation before the Coronavirus outbreak”.

Part two of the questionnaire contained 21 additional questions related to the secondary objectives of the study. Participants could choose to skip this section. In this section, physical functioning was assessed with the corresponding domain of the EORTC QLQ-C30 questionnaire [27], using a short-form selection from the item-response theory-based EORTC item bank. The short form was selected to best cover the expected levels of functioning of the target population. Items were measured on a 4-point scale, which was linearly transformed to a 0–100 score using proprietary scoring software of the EORTC. Higher scores reflect a better level of functioning, with a score of 50 representing an average level of physical functioning for an age- and gender-matched sample from a European normative sample for the general population. Fatigue was assessed using the Abbreviated Fatigue Questionnaire [28], which consists of four items scored on a 7-point Likert scale. Sum scores were calculated following the published scoring algorithm and had a possible range from 4 to 28 [28]. Presence of comorbidity was assessed using a modified version of the Charlson Comorbidity Index [29]. Items on current level of PA included the number of days in an average week on which respondents engaged in muscle-strengthening activities and aerobic activities of at least moderate intensity. These items were also used to classify respondents as meeting the exercise guidelines or not. Self-rated knowledge and skills for exercising were posed using 4 statements (e.g. “I know how much exercise I should do to improve my health”) with a 5-point Likert-type response scale.

Experienced barriers to exercising, in general, were predefined and selected from the literature. Respondents could respond on a 5-point adjective rating scale.

Attitudes towards exercise were assessed using an 11-point scale with adjective anchors and included instrumental attitudes (useful-useless, beneficial-harmful, and wise-foolish) and affective attitudes (pleasant-unpleasant, interesting-boring, and easy-hard), as used in previous studies [30, 31]. Higher scores indicate a more positive attitude towards exercise.

Finally, respondents were asked to select their three most important personal goals for taking part in an exercise programme from a list that also included an “other, namely...” option and to indicate other supportive interventions that they thought could be complementary or alternatives to an exercise intervention.

### Data collection and statistical analysis

All data were captured using Electronic Case Report Forms (eCRFs) in Qualtrics (Qualtrics, Provo, UT), which is a fully GDPR-compliant and ISO 27001-certified survey system, approved for research by the Amsterdam University of Applied Sciences*.* Data were imported into R 4.1.1. statistical software for analysis [32].

Descriptive statistics were generated to characterise the sample and to summarise the responses with regard to experience with advice, attitude, preferences, barriers, fatigue, quality of life, awareness about the importance of exercising, personal goals, and supportive interventions.

To investigate correlates of being counselled about exercise during cancer care and the correlates of a preference for supervised exercise, we used multivariable logistic regression models. For each outcome variable, we first tested candidate predictors based on theoretical considerations in univariable regression models. Variables with a univariable beta-coefficient *p*-value < 0.20 were entered into a multivariable logistic regression model, and backward selection was applied to select the most strongly correlated variables for the final models. Variables were retained at *p*-values < 0.05. Results of the regression analyses are reported as odds ratios with 95% confidence intervals and accompanying *p*-values, and Nagelkerke’s *R*-squared as an approximation of explained variance.

As explanatory variables for exercise being discussed in the context of cancer care, we considered patients’ age, education level (high: bachelor’s degree, master’s degree, or doctoral degree; middle: secondary vocational education; low: no education, primary education, and general secondary education), comorbidity (none, one, or more than one), and type of treatment. The latter was dichotomised into hormonal therapy (yes vs no) since current guidelines for treatment of PC explicitly mention the need to discuss exercise when hormonal therapy is used [21], and the sample size did not allow for further subclassification into smaller treatment groups. Given the increasing awareness that exercise should be part of cancer care, we also explored whether patients who were treated more recently also received exercise advice more often.

For the analysis of a preference for supervised exercise, variables of interest were age, educational level (high, middle, or low), being insured for exercise programming, treatment intent (curative, palliative, or active surveillance), comorbid conditions (none, one, or more than one), exercise behaviour (meeting Dutch guidelines for the general population versus not meeting guidelines), total barrier score, fatigue score, and physical functioning score.

Missing data were judged to be missing completely at random based on a nonsignificant Little’s MCAR test and absence of correlation of response status with the outcome variables.

However, complete case analysis discarded 17% of the data for the regression models. We therefore performed sensitivity analyses using multiple imputations. For these analyses, we generated 20 imputed datasets using fully conditional specification and pooled results using Rubin’s rules (mice package in R) [33].

## Results

In total, 171 patients with PC completed the first section of the questionnaire, of whom 153 patients completed the second section. Participant characteristics are presented in Table 1. The respondents had a mean age of 70 years (SD = 6.5), and over half had a college or university degree (55%). Respondents were from all twelve provinces of the Netherlands, with variation in participation across provinces largely reflecting differences in population density. The median time since diagnosis was two years (IQR 1–5). Thirty-nine percent of the patients received surgery as first treatment, 31% systemic therapy, including hormonal therapy, 12% brachytherapy, 14% external beam radiation therapy, and 13% other treatments^*^. Thirty-six percent of the 58 respondents who received additional treatments after their first treatment also underwent hormonal therapy. The majority of the respondents (61.6%) reported one or more comorbidities, with hypertension and arthrosis being the most prevalent health problems. The mean physical functioning summary score was 50.6 (SD = 8.67), and the median fatigue score was 8.5 (IQR 5–14.8).

**Table 1:** Descriptive statistics of participants’ characteristics

	Participants (*N* = 171)
Age in years, *M* (SD)	70.1 (6.5)
Educational level, *N* (%)	
*No education*	1 (0.6)
*Primary/middle school*	25 (14.7)
*High school*	47 (27.5)
*College/university*	94 (55.0)
*Other*	4 (2.3)
Time since first treatment in years, median (IQR, missing)	2 (1–5, 8)
First treatment^1^, *N* (%)	
*Surgery*	66 (38.6%)
*Brachytherapy*	20 (11.7%)
*External beam radiation therapy*	54 (13.6%)
*Systemic treatment*	53 (31.0%)
*Other*	22 (12.9%)
Additional treatment^1^, *N* (%)	
*Surgery*	6 (10.3%)
*Exterior radiation therapy*	22 (38.0%)
*Systemic treatment*	21 (36.2%)
*Treatment for osteoporosis*	5 (8.6%)
*I don’t know exactly*	3 (5.2)
*Other*	21 (36.2%)
Treatment intention, *N* (%)	
*Curative*	85 (49.7%)
*Palliative*	70 (40.9%)
*Active surveillance*	10 (5.8%)
*Unknown*	6 (3.5%)
Physical functioning^2^, mean (SD)	50.6 (8.7)
Fatigue level^3^, median (IQR)	8.5 (5–15)

^1^Percentages count up above 100% due to multiple answer options; ^2^range 0–100, higher score indicates better physical functioning; ^3^range 4–28, higher score indicates higher levels of fatigue

### Referral and advice

Of all participants, 56% reported never having been asked about their current level of PA in the context of cancer care. Moreover, 65% reported never having been educated about possibilities and benefits of exercise at any time during their cancer treatment or follow-up. When provided, advice was mostly given in the form of a conversation (*n* = 46, 78%) or information leaflet (*n* = 14, 24%)^*^. Sixty-five percent reported never having been adviced to do more exercise or having received a referral to an exercise programme at any point during or after treatment. Participants who did recall such advice mostly received this from nurses/nurse practitioners (*n* = 28, 47%), followed by hospital-based physicians (*n* = 19, 32%), physical therapists (*n* = 13, 22%), or general practitioners (*n* = 10, 17%)^7^.

### Preferences for exercise programs

Preferences for exercise programmes are summarised in Table 2. Walking (65%), cycling (64%), and strengthening exercises (43%) were the most preferred exercise modalities. Outdoor exercise (33%) or exercise in a fitness centre setting (28%) were the most commonly endorsed settings. Forty-nine percent of the respondents would like supervision during exercising, preferably from a physiotherapist (24%) or fitness instructor (19%). Half of the patients indicated a preference for exercising in a group, while the other half preferred to exercise alone. For those who preferred group exercise, exercising with other people from the general public (26%) was preferred over-exercising with other cancer patients (11.7%), family (7%), or friends (6.4%). Preferences for frequency of exercise varied, with 27% preferring twice a week, 19% more than three times a week, and another 19% once per week, while the remaining 35% had a different or no preference. Regarding exercise intensity, the large majority (72%) preferred a moderate intensity level. Finally, the modal acceptable travel time was 10–20 min, and very few respondents (2.3%) were willing to travel > 30 min for an exercise programme.

**Table 2:** Preferences for exercise programmes

	*N* (%)
Preferences of the group to exercise with^1^ (*N* = 160)	
*Other people in general*	45 (28.1%)
*With no one, alone*	42 (26.2%)
*No preference*	25 (15.6%)
*Other men with PC*	12 (7.5%)
*Family*	12 (7.5%)
*Friends*	11 (6.9%)
*Other people with cancer*	8 (5%)
*I don’t know*	5 (3.1%)
Preferences for supervision^1^ (*N* = 160)	
*Physiotherapist*	41 (25.6%)
*No one*	41 (25.6%)
*Fitness instructor*	32 (20.0%)
*No preference*	23 (14,4%)
*I don’t know*	12 (7.5%)
*Other health professional*	5 (3.1%)
*Peer counsellor*	3 (1.9%)
*Nurse practitioner*	3 (1.9%)
Preference for location for exercising^1^ (*N* = 160)	
*Outdoor*	52 (32.5%)
*Fitness centre*	44 (27.5%)
*Home*	22 (13.8%)
*Physiotherapists practice*	19 (11.9%)
*No preference*	11 (6.9%)
*Oncology centre*	10 (6.2%)
*I don’t know*	2 (1.2%)
Time willing to travel to exercise programme ^1^ (*N* = 157)	
*Less than 10 min*	38 (24.2%)
*10–20 min*	79 (50.3%)
*20–30 min*	23 (14.6%)
*More than 30 min*	4 (2.5%)
*I don’t know*	13 (8.3%)
Length of time able to be physically active ^1^ (*N* = 160)	
*Less than 10 min*	5 (3.1%)
*10–20 min*	14 (8.8%)
*20–30 min*	22 (13.8%)
*30–45 min*	32 (20.0%)
*More than 45 min*	75 (46.9%)
*I don’t know*	12 (7.5%)
Preference for intensity of exercise^2^ (*N* = 160)	
*Mild intensity*	15 (8.8%)
*Moderate intensity*	123 (71.9%)
*High intensity*	36 (21.1%)
*No preference*	3 (1.8%)
Preference for frequency of exercise ^1^ (*N* = 160)	
*Occasionally a session*	2 (1.2%)
*Once every 2 weeks*	3 (1.9%)
*Once a week*	32 (20.0%)
*Twice a week*	46 (28.7%)
*Three times a week*	28 (17.5%)
*More than three times a week*	33 (20.6%)
*No preference*	5 (3.1%)
*I don’t know*	11 (6.9%)
Maximum amount willing to pay for exercise program^1^ (*N* = 158)	
*Nothing*	14 (8.9%)
*Up to 5 euros*	3 (1.9%)
*Up to 15 euros*	32 (20.3%)
*Up to 25 euros*	60 (38.0%)
*Up to 50 euros*	21 (13.3%)
*Up to 75 euros*	4 (2.5%)
*Up to 100 euros*	3 (1.9%)
*I don’t know*	21 (13.3%)
Preferences for type of exercise^3^, *N* (%)	
*Walking*	111 (64.9%)
*Cycling*	110 (64.3%)
*Muscle strengthening*	74 (43.3%)
*Circuit training*	42 (24.6%)
*Flexibility training*	33 (19.3%)
*Aerobic exercise*	33 (19.3%)
*Swimming*	28 (16.4%)
*Mind-body exercise*	22 (12.9%)
*Other*	18 (10.5%)
*Other sports and/or game activities*	14 (8.2%)
*Bootcamp*	6 (3.5%)
*No preference*	4 (2.3%)

^1^One option possible; ^2^multiple options possible; ^3^chosen as one of three activities most interested in

### Reimbursement and willingness to pay

The majority (69%) did not know whether their insurance covered exercise programmes for people with cancer in the context of illness or rehabilitation. Only 7% reported that all programmes were covered by their insurance, while 14% reported that it was reimbursed only under specific conditions. Mixed preferences were shown for willingness to pay for an exercise programme, with a range between 5 and 100 euros per month, a mode of 25 euros per month, and only 4% being willing to pay over 50 euros per month.

### Outcome expectations and values

Full details of reported outcome expectations and values are provided in Table 3. In general, most respondents had moderately positive outcome expectations about the effects of PA and indicated that these anticipated effects were important to them. The median expectation*value was 23 (IQR: 14–29). Muscle strength and bone strength were expected to improve most strongly. Sleeping well, having a positive mood, and good bone health were the highest valued outcomes.

**Table 3:** Outcome expectations (range − 5 to 5)^1^ and values (range 0–10)^2^

	Mean (SD)
Muscle strength	
*Expectancy*	3.59 (1.40)
*Value*	8.21 (1.42)
Self-esteem	
*Expectancy*	2.55 (1.82)
*Value*	7.60 (1.81
Mood	
*Expectancy*	2.85 (1.28)
*Value*	8.59 (1.13)
Sleep	
*Expectancy*	2.42 (1.35)
*Value*	8.62 (1.04)
Stress	
*Expectancy*	2.61 (1.50)
*Value*	8.01 (1.61)
Fatigue	
*Expectancy*	2.50 (1.66)
*Value*	8.26 (1.40)
Pain	
*Expectancy*	1.33 (2.14)
*Value*	7.22 (2.51)
Strength of bones	
*Expectancy*	3.09 (1.24)
*Value*	8.51 (1.14)

^1^Negative scores represent the expectation that exercise will worsen the outcome, positive scores that the outcome will improve; ^2^Higher scores indicates a higher personal value for the respective outcome

### Attitudes and barriers

**Fig. 1:**
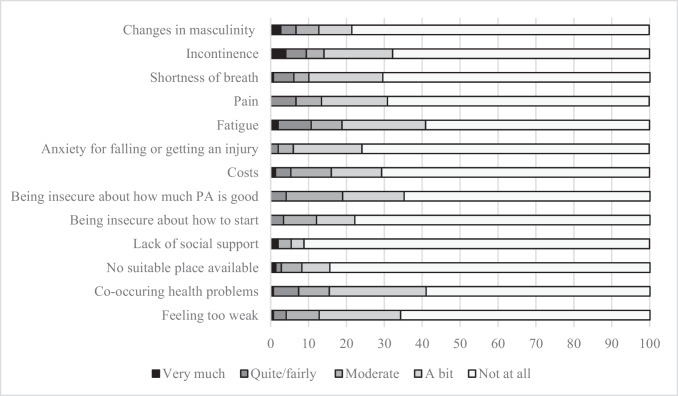
Barriers to be physically active, reported by 149 PC patients

Overall, participants had positive instrumental and affective attitudes towards exercise and regarded it as useful (*M* = 4.35, SD = 0.98), beneficial (*M* = 4.16, SD = 1.53), pleasant (*M* = 3.54, SD = 1.54), interesting (*M* = 3.44, SD = 1.58), easy (*M* = 2.61, SD = 1.85), and wise (*M* = 4.3, SD = 1.00). At the same time, 113 respondents (74%) reported experiencing at least some barriers to being physically active, of whom 49% reported a barrier that was at least moderate. The most frequently reported barriers were fatigue, endorsed by 39.2%, and health problems other than PC, endorsed by 38.6% (Fig. 1).

### Associations with being counselled on exercise

In the complete case analysis, of the variables under consideration, receiving hormonal therapy and time since treatment were retained as predictors of having received counselling regarding exercise (OR = 4.6, 95% CI [2.1, 11], *p* < 0.001, OR = 0.89, 95% CI [0.79, 0.98], *p* = 0.03). Using the imputed data, receiving hormonal therapy and age were retained, with hormonal therapy increasing the likelihood of having received counselling (OR = 4.2, 95% CI [2, 8.7], *p* < 0.001) and older age decreasing the likelihood (OR = 0.93, 95% CI [0.88, 0.99], *p* = 0.01), while time since treatment was no longer significant. Nagelkerke’s *R*-squared for the model including hormonal therapy and age, using all available data, was 16%.

### Associations with preference for supervision

In the multivariable analysis of preference for supervision, physical functioning was discarded because of high collinearity with fatigue. Being insured appeared to be the major driver for preferring supervised exercise (OR = 6.3, 95% CI [2.2, 22.9], *p* = 0.002). Of the patient variables of interest, only the fatigue score contributed significantly to the preference for supervision in the final multivariable model, with higher fatigue increasing the likelihood of preferring supervision. (OR = 1.09, 95% CI [1.02, 1.17], *p* = 0.010). Together, insurance and fatigue explained 11% of the variability in supervision preference using all available data. Sensitivity analysis after multiple imputations yielded similar results.

## Discussion

Our goals in this study were to gain insight into the experiences of patients with PC with regard to exercise advice in the context of health care, to assess their preferences for and outcome expectations of an exercise programme after a PC diagnosis, and to explore their general attitudes and perceived barriers. In addition, we explored factors associated with receiving exercise counselling and preferring supervision.

We found that the majority of the respondents did not recall ever having been asked about their current level of PA or adviced about possibilities and benefits of exercise in the context of their cancer care. This finding is similar to previous studies in which less than half of oncologists reported having given such advice [34–36]. Since we relied on patient self-report, it is unclear whether this advice was indeed not provided or that patients did not recall it (which could also indicate that the information provision was not effective). A previous study found discrepancies between what healthcare professionals and patients report [36]. The fact that participants who received hormone therapy were more likely to recall having received exercise advice is in line with recommendations of the EAU guidelines for PC [21].

Nevertheless, even among patients on hormone therapy, a substantial proportion did not recall having received exercise advice. Although in univariable analysis a longer time since treatment was associated with lower likelihood of being counselled for exercise, this association was weaker and no longer statistically significant in the multivariable analysis. These results suggest that the guideline recommendations have not yet been translated adequately into clinical care pathways. Clinicians are adviced to “assess, advice, and refer (as appropriate” at repeated intervals during cancer treatment [16]. In what form advice should be given to be most effective, and what constitutes optimal timing in relation to, for example, teachable moments, requires further research.

Older patients had significantly lower odds of receiving exercise advice, which we hypothesize may reflect a subjective judgement of health care professionals about patients’ (in)ability to engage in structured exercise. As patient factors only explained 16% of the variance, it is likely that healthcare professional factors such as perceived lack of competence to discuss it and healthcare contextual factors such as lack of available time also contribute to practice variation in this regard, as has been reported in previous studies in mixed cancer populations [34, 37].

Walking, cycling, or strengthening exercises were the most preferred exercise modalities, which is comparable to preferences reported in previous research in mixed cancer populations as well as among PC survivors specifically [23, 38]. This underscores the need for diverse and flexible exercise programming for people living with and beyond PC. Only 11.7% preferred exercising with other people with cancer or PC, although this has previously been highlighted as an important factor for exercise enhancement [2].

Almost half of the respondents would prefer supervision during exercising, typically by a physiotherapist or fitness instructor, while only 16% were prepared to travel more than 20 min for their exercise programme. Although there undoubtedly are sociocultural and geographical differences regarding what is considered acceptable in absolute terms, travel time has been recognised as an important barrier to uptake of physical exercise in other patients with cancer [31, 39–41]. In the Netherlands, a dense network of physiotherapists with sufficient knowledge of cancer (Onconet) is available. These physiotherapists are situated within 20 min travel time from almost any address in the Netherlands. Further, there are ample possibilities for gym-based exercise within a 20-min radius, and the number of exercise professionals with oncology certification is increasing rapidly. Travel time is therefore not likely to impede uptake of exercise programmes by people with PC in the Netherlands.

Many respondents indicated a preference for exercising outdoors. This is comparable to the findings of previous studies [38, 42]. Current exercise programming for cancer survivors in the Netherlands does not align with these preferences, as exercise for this population is typically provided in physiotherapy practices or gym settings. Post hoc analysis showed that, of the patients with a preference for supervision, only 16% preferred outdoor exercise. Still, attention to developing outdoor exercise programming for people with PC is needed to accommodate those who prefer outdoor exercise.

Costs presented a barrier, even though only 29% of patients reported them as such. Willingness to pay among the respondents was generally low, and often too low to cover physical therapy expenses. Although all people in the Netherlands have basic healthcare insurance, this does not cover physical therapy and exercise programmes. Supplemental insurance packages are available that provide different levels of reimbursement for exercise or physical therapy, but not all people have such supplemental insurance. The lack of reimbursement from basic insurance likely limits the uptake of exercise for people with PC who require supervision in a health care setting. Such supervision would be required, at least initially, for patients with bone metastases at high risk of falling or having risks or limitations related to comorbid conditions [16].

Preferences regarding exercise frequency, modality, and intensity were largely consistent with current recommendations for people with PC [2]. Interestingly, these preferences did not reflect the respondents’ actual current PA levels; most participants did not meet the current PA guidelines.

The fatigue score of the respondents was lower compared to the available reference values for the shortened fatigue questionnaire for people with cancer [28]. Also, their physical functioning level was comparable to the score of an average age- and gender-matched European person (*M* = 50, SD = 10). Still, fatigue was among the reported barriers to exercise uptake and a significant predictor of preferring supervision. Fatigue and other health problems reported as barriers to exercise by PC patients in this study as well as in previous studies [25, 38] are known to respond well to exercise [2]. These findings underscore the need for better exercise counselling, especially considering the relatively low outcome expectancy scores, which indicate that patients might not be fully aware of the health benefits of exercise. Also, lack of knowledge about exercise (i.e. not knowing how much exercise is needed to achieve health benefits or not knowing how to start exercising) was reported quite frequently as a barrier and could be fairly easily addressed by developing and distributing good educational materials, in print or using multimedia. Such materials could also be used to improve outcome expectancies. For some patients, a single physical therapy consultation might suffice to reduce uncertainties and provide informed advice.

Although our study provides useful insights that can help to implement exercise as a regular part of health care for patients with PC, some limitations should be noted. There is a potential risk for selection bias with an overrepresentation of patients who are relatively exercise-minded. Also, the respondents’ education level was relatively high. This could limit the generalizability of our findings. Moreover, as with all questionnaire research, there may have been a recall bias because of retrospective self-report, the use of predefined lists with limited response choice, and Likert-scale-type questions limited the opportunity for in-depth exploration of preferences. Also, the preferences for attributes of exercise programming identified in this study were limited to single attributes. Future research could quantify preferences for combinations of attributes by means of, for example, discrete choice experiments. Most questions used in the first part of the survey were study-specific. Therefore, evidence on (construct) validity of these items is lacking. For study-specific items that were used as multi-item scales, we calculated internal consistency reliability, which was adequate in all cases. Finally, despite our request to respond to the questions considering their pre-pandemic situation, the COVID-19 outbreak may still have influenced participants’ responses to the survey questions, for example when considering the benefits or drawbacks of exercising outside or in the company of others. The study also has notable strengths, including the inclusion of patients undergoing palliative treatment, the low risk of social desirability bias due to the anonymous data collection, and the detailed information obtained on exercise counselling in the context of PC care.

In conclusion, the limited recall of exercise counselling by the survey respondents indicates that despite professional guideline recommendations, exercise is not sufficiently discussed in clinical practice. This highlights the need to improve patient education about the benefits of exercise for people with PC. People with PC experience various barriers that limit them from exercising. Although they are open to supervised exercise and expect exercising to improve their health, their awareness of potential benefits is still limited. Finally, due to the low willingness to pay, the current lack of reimbursement for physical therapy services in the Netherlands may limit the uptake of exercise programmes for those patients in need of such services. These findings can contribute to the development and implementation of successful exercise programmes for people with PC.

## Data Availability

The datasets generated during and/or analysed during the current study are available from the corresponding author upon reasonable request.
